# Biologic therapy for autoimmune diseases: an update

**DOI:** 10.1186/1741-7015-11-88

**Published:** 2013-04-04

**Authors:** Ziv Rosman, Yehuda Shoenfeld, Gisele Zandman-Goddard

**Affiliations:** 1 Department of Medicine C, Wolfson Medical Center, 61 Halochamim Street, POB 63, Holon, 58100 Israel; 2 Zabludowicz Center for Autoimmune Diseases, Sheba Medical Center, Sheba 2, Ramat-Gan, Israel; 3 Sackler Faculty of Medicine, Tel-Aviv University, Tel-Aviv, Israel

**Keywords:** Biologics, Anti-TNF, B cell depletion, Autoimmune diseases, Rheumatoid arthritis, Spondyloarthritis, Systemic lupus erythematosus, Systemic sclerosis, Vasculitis

## Abstract

Biologic therapies for rheumatologic diseases, which are targeted at molecules involved in the mechanisms of the immune system, provide an alternative to the existing treatment methods of disease-modifying anti-rheumatic drugs and other immunosuppressive medications. However, the current drawbacks of biologic therapies, including the inconvenience of intravenous administration, the high costs of these drugs, and the adverse events associated with them, prevent their wide use as first-line medications. This review provides an update of the recent literature on the new biologic therapies available. The review concentrates on nine drugs: tocilizumab, rituximab, ofatumumab, belimumab, epratuzumab, abatacept, golimumab, certolizumab, and sifalimumab, which are used as therapies for rheumatoid arthritis, spondyloarthritis, systemic lupus erythematosus, systemic sclerosis, or vasculitis.

## Introduction

The use of biologic therapies as an adjunct to disease-modifying anti-rheumatic drugs (DMARDs) for the treatment of autoimmune and rheumatologic diseases is rapidly expanding, owing to the good efficacy and safety profiles of these drugs, and the better understanding of the initial targets of altered immune regulation and activity in various diseases. Targeted therapies such as these are often well tolerated by patients. However, the inconvenience of intravenous (IV) administration, as well as the high costs and adverse events (AEs) associated with these drugs prevent their wide use as first-line medications. The major targets of most biologic therapies are cytokines, B cells, and co-stimulation molecules. Anti-cytokines include anti-tumor necrosis factor (TNF)-α, anti-interleukin (IL)-1, and anti-IL-6 molecules. B-cell depletion includes use of anti-CD20 antibodies and B cell receptor (BCR) modulation by the B-lymphocyte stimulator (BLyS). Although some of the biologic therapies have been found to be useful in more than one disease, others are specific for a single disease. Research is ongoing to identify other molecular targets.

In this review, we provide an update on some of the new agents that have become available in the past 5 years for clinical treatment of rheumatoid arthritis (RA), spondyloarthropathy, systemic sclerosis (SyS), systemic lupus erythematosus (SLE), and vasculitis.

## Methods

We performed a thorough literature review of all papers in English published in PubMed during the period 1 January 2007 to 30 April 2012. We matched the terms: ‘rheumatoid arthritis’, ‘spondyloarthropathy’, ‘systemic sclerosis’, ‘systemic lupus erythematosus’, and ‘vasculitis’ with the terms ‘biologics’, ‘tocilizumab’, ‘rituximab’, ‘ofatumumab’, ‘belimumab’, ‘epratuzumab’, ‘abatacept’, ‘golimumab’, ‘certolizumab’, and ‘sifalimumab’. Reports of randomized controlled trials (RCTs) and case series were included. Case reports and any reports of biologic therapies that are not yet available for clinical use were excluded. We excluded articles that were in a language other than English.

### Screening for biologic treatment

Over a decade has passed since the introduction of biologic therapies for autoimmune diseases. Currently, screening is routine practice prior to administration of these drugs, and is often performed during the initial visits to the outpatient clinic to prevent unnecessary waits for the patient when a biologic is indicated. Screening consists of evaluation for previous or current tuberculosis (TB) infection (based on history, purified protein derivative (PPD) test, chest radiography), serological evidence of hepatitis B and C, history of malignancies or neurological disease. Based on the screening results, the physician will assess which biologic treatment is recommended or if prior treatment is warranted before the initiation of the biologic therapy. The choice of biologic agent for rheumatologic diseases is then tailored to the patient's needs and lifestyle.

### Tocilizumab

#### Mechanism

Tocilizumab (TCZ; trade names Actemra, Roactemra) is a recombinant monoclonal IgG1 anti-human IL-6 receptor (IL-6R) antibody (Table 1) [1]. IL-6 binds to either membrane-bound or soluble IL-6R, and this complex in turn binds to the 130 gp signal transducer. This process enhances the inflammatory cascade, inducing angiogenesis and amplifying the activity of adhesion molecules and the activation of osteoclasts [2,3]. IL-6 is also responsible for activating both T and B helper cells, and is involved in B-cell differentiation, thus by blocking IL-6, the inflammatory response is decreased [2].

**Table 1:** Update on biologic therapy in autoimmune diseases

**Drug (trade name)**	**Mechanism of action**	**Indications**
Tocilizumab (Actemra)	Recombinant monoclonal IgG1 anti-human interleukin 6-receptor antibody [1]	RA after treatment failure with anti-TNF,^1 ^SJIA^1^[5-7]
Rituximab (Rituxan)	Chimeric human monoclonal antibody against the CD20 protein [12]	RA,^1 ^WG, MPA. Off-label use: ITP, refractory pemphigus vulgaris [13-23]
Ofatumumab (Arzerra)	Fully human monoclonal antibody directed against membrane proximal epitope on the CD20 molecule [25]	RA,^1^[24,25]
Belimumab (Benlysta)	Human monoclonal immunoglobulin IgG1 gamma, which binds to and inhibits the soluble form of the BLyS protein [27,28]	SLE^1^[26,27]
Epratuzumab (Lymphocide)	IgG1 monoclonal antibody directed against the CD22molecule [29,30]	SLE, Sjögren’s syndrome [28-30]
Abatacept (Orencia)	CTLA-4 IgG1 binding to CD80/86 on antigen-presenting cells inhibiting the co-stimulation of CD28 on the T cells [32]	RA,^1 ^JIA,^1^ SLE – discoid, serositis and arthritis manifestations [31-35]
Golimumab (Simponi)	IgG1 monoclonal antibody, acting on TNF-α, both soluble and membrane-bound [37]	RA,^1^ PsA,^1^ AS^1^[36-40]
Certolizumab (Cimzia)	Pegylated humanized antibody Fab^′ ^fragment of the TNF-α monoclonal antibody [42]	RA^1^[41,42]
Sifalimumab	An anti-IFN-α monoclonal antibody [44]	Phase III trial [43,44]
Intravenous immunoglobulin	Pool of immunoglobulins from healthy individuals. Many mechanisms involved [48,49]	SLE, systemic sclerosis, vasculitis [49,50]
Anakinra (Kineret), canakinumab (Ilaris), rilonocept (Arcalyst)	All three are interleukin-1 blockers	RA, CAPS [45-47]

^1^Approved by the US Food and Drugs Administration. Abbreviations: AS, ankylosing spondylitis; BLyS, B-lymphocyte stimulator; CAPS, cryopyrin-associated periodic syndrome; CTLA, cytotoxic T lymphocyte-associated; JIA, juvenile idiopathic arthritis; MPA, microscopic polyangiitis; PsA, psoriatic arthritis; RA, rheumatoid arthritis; SLE, systemic lupus erythematosus; TNF, tumor necrosis factor; Wegener’s granulomatosis.

In patients with RA, a high level of IL-6 is present in the blood and in the synovium of involved joints. In an animal study, injecting TCZ into the inflamed joints reduced the swelling and the inflammatory response [2,4].

#### Indications and dosage

TCZ is indicated for the treatment of RA following an inadequate response or treatment failure with DMARDs or TNF alpha antagonists (anti-TNF alpha drugs). It is also indicated as first-line therapy for patients with severe systemic juvenile idiopathic arthritis (SJIA) and for Castleman's disease (Table 1).

The recommended dose of TCZ is 8 mg/kg every 4 weeks. The drug was approved for RA in January 2010 in the USA, but the US recommendations are for a starting dose of 4 mg/kg once every 4 weeks, followed by an increase to 8 mg/kg depending on clinical response [5,6]. The route of administration is IV, with the dose of 4 to 8 mg/kg IV administered as a single infusion every 4 weeks for RA and 12 mg/kg or 8 mg/kg IV (depending on body weight) for SJIA [6]. Within an RA population, in Disease Activity Score(DAS) remission rate was 55.3% for patients treated for 5 years on monotherapy [7].

#### Efficacy

A meta-analysis examined published articles on double-blind, randomized, placebo-controlled trials that indirectly compared TCZ with one or more of the following biologics: abatacept, rituximab, or anti-TNF-alpha blockers (etanercept, infliximab, and adalimumab), in patients with inadequate response to DMARDs and/or anti-TNF-alpha blockers. TCZ was non-inferior compared with the other biologic therapies according to the American College of Rheumatology (ACR) criteria for a 20% (ACR20) or 50% (ACR50) improvement, and was superior for a 70% improvement (ACR70) [8]. Furthermore, the response to TCZ occurred early, soon after the first infusion [1].

Monotherapy with TCZ for 52 weeks resulted in significantly reduced radiographic change (total Sharp score) compared with DMARDs [2]. In a 24-week study comparing TCZ and methotrexate (MTX), TCZ was found to be non-inferior to MTX in the first week and superior to MTX in the second week in the intention-to-treat group, as measured by ACR20 [3]. Several other studies comparing monotherapy of MTX with that of TCZ have also shown superiority of TCZ [3,9]. In a study of 1,196 patients with RA who responded partially to MTX, treatment with TCZ led to suppression in radiographic progression and improvement in physical function [9]. Other studies reported response to TCZ in patients with RA who failed to respond to anti-TNF-alpha blockers [9].

#### Adverse effects and safety

Favorable safety results for TCZ were reported for both short-term and long-term treatment of moderate to severe RA. In one meta-analysis, TCZ was well tolerated for more than 2.4 years of treatment, and the AEs were less severe compared with other biologic therapies [8]. In a 24-week study of 286 patients with RA, 66.1% experienced AEs related to the drug, which were mild to moderate and transient. A small number of patients experienced serious AEs, which were predominantly infections [1].

In a study integrating the three phases of TCZ safety, the AEs were similar to the other treatment groups (DMARDs or anti-TNF-alpha). The most common AEs were infections, mostly of the upper respiratory tract (URTI) and gastrointestinal (GI) tract [10]. More severe AEs included cardiac events, serious infections, solid-organ malignancies, non-melanoma skin tumors, and hematological disturbances [10]. Higher rates of serious infections were related to previous anti-TNF-alpha treatment. The most common infections were pneumonia, gastroenteritis, and urinary-tract infections [10]. Some patients were diagnosed with TB despite being screened before treatment in accordance with the guidelines. Higher doses of TCZ (8 mg/kg) were associated with higher risks for infection, but rates were still similar to those encountered with DMARDs or anti-TNF-alpha blockers [10]. GI perforation occurred in 16 patients (predominantly women) exposed to TCZ in the phase III trials, with 11 of them developing diverticuli [10]. Some patients developed a significant increase in liver-function tests, indicating liver dysfunction; a dose reduction was sufficient for the continuation of the study. Only 2.3% of TCZ-exposed patients had to discontinue treatment because of liver abnormalities [10]. There was a reduction in neutrophil count in patients receiving TCZ, which stabilized after 2 weeks of therapy. Some patients developed grade 4 neutropenia, but the neutrophil count normalized after discontinuation of therapy [10].

In other studies of monotherapy with TCZ, the AEs reported were nasopharyngitis, GI symptoms, and infections. There was no difference in the incidence of AEs with TCZ compared with anti- TNF-alpha blockers [3]. TCZ was associated with increases in cholesterol levels and in the ratios of low-density lipoprotein (LDL) to high-density lipoprotein (HDL) cholesterol, and of total to HDL cholesterol [11].

In conclusion, TCZ is beneficial and safe for treatment of RA in cases of non-response to anti-TNF-alpha therapy or when anti-TNF-alpha therapy is contraindicated.

### Rituximab

#### Mechanism

Rituximab (trade names Rituxan, Mabthera) is a chimeric human monoclonal antibody against the CD20 protein found on naive, mature, and memory B cells. Rituximab depletes the B-cell population via apoptosis, cellular cytotoxicity, and complement activation. In a number of studies measuring markers for immature B cells, memory B cells, and pre-B cell colony-enhancing factor (visfatin), B-cell depletion occures after treatment with rituximab [12]. In addition, rituximab affects the interferon (IFN) I response genes. In patients with RA responding to rituximab treatment, expression of IFN response genes (*RSAD2*,*IFNI44L*, *HERC5*, *LY6E*, *Mx1*) increased, whereas the non-responding patients had limited or no IFN gene-expression activity (Table 1) [12].

#### Indications and dosage

For autoimmune diseases, the only indication for which rituximab is approved by the Food and Drugs Administration (FDA) is active RA unresponsive to DMARDs and anti-TNF-alpha agents. Rituximab is beneficial for other off-label indications in patients with autoimmune disease (such as SLE) and Castleman's disease (Table 1).

The most popular protocol for RA is IV infusion of 1000 mg/m^2^ on days 1 and 15 in combination with MTX. Subsequent courses may be administered every 24 weeks (based on clinical evaluation), and if necessary, may be repeated, but no sooner than every 16 weeks. For patients with RA, pre-medication with IV methylprednisolone 100 mg (or equivalent) is recommended, 30 minutes before each dose of rituximab [13]. For granulomatosis with polyangiitis (GP) (previously Wegener's granulomatosis), the protocol is different: IV infusion with 375 mg/m^2^ once weekly for four doses (in combination with IV methylprednisolone for 1 to 3 days followed by daily prednisone). The protocol for microscopic polyangiitis (MPA) is similar to that of GP [14].

Rituximab was evaluated for chronic immune thrombocytopenic purpura (ITP) in a multi-center phase II study of 60 patients, who received an IV infusion of 375 mg/m^2^ once weekly for 4 doses; 40% of patients achieved a steady platelet level [15]. A few studies also suggested that low-dose treatment with 100 mg/m^2^, either alone or in combination with steroids would suffice and result in fewer AEs; however, further data on this dose are lacking [15].

For refractory pemphigus vulgaris (PV), the recommended treatment is IV infusion of 375 mg/m^2^ rituximab once weekly in weeks 1, 2, and 3 of a 4-week cycle, which is repeated for one additional cycle, followed by one dose per month for 4 months (total of ten doses in 6 months) [16]. The initial infusion should be started at 50 mg/hour, and if there is no reaction, the rate should be increased by 50 mg every 30 minutes (100 mg/hour).

#### Efficacy

Several studies suggest that rituximab may be beneficial for the treatment of SyS [17]. In a study of eight patients with SyS, the B-cell infiltrate was depleted in the skin after rituximab infusion, signifying that the drug might be a possible therapy for skin fibrosis [17]. Another study of 15 patients with SyS also showed histological improvement in the skin after rituximab therapy [17].

In a study of 257 patients with SLE treated with rituximab and prednisone, disease activity was not significantly improved compared with placebo. However, in a subgroup analysis of African American and Hispanic patients, there was a significant benefit for rituximab therapy. Furthermore, in open trials for long-term treatment, rituximab was found to be superior [18]. The lack of efficacy in the overall trial could be associated with the clinical set-up of the trial, inclusion of too many subsets, or non-stratification of patients positive or negative for anti-double-stranded DNA antibodies [19].

A case series indicated that rituximab might be beneficial in hemolytic anemia, thrombocytopenia, and arthritis-related SLE [20].

In a study of 646 patients with RA who had treatment failure with anti-TNF-alpha blockers, follow-up at 6 months after therapy with rituximab resulted in a good clinical response and even remission of the disease [21].

In the SUNRISE (Study of Retreatment with Rituximab in Patients with Rheumatoid Arthritis Receiving Background Methotrexate) trial, 559 patients with RA with inadequate response to one or more TNF-alpha inhibitors were given two treatment cycles of rituximab to assess the efficacy and safety profile of the drug. Of the total 559 patients in the study, 475 patients received the second cycle of therapy, with a significant response compared with the placebo group, as measured by the ACR20 [13].

In a study of 42 patients with severe PV, rituximab was administered as monotherapy, inducing remission in 36 patients for periods of 8 to 64 months. In those patients requiring an additional dose, the safety profile remained good [16].

#### Adverse effects and safety

One of the AEs associated with rituximab is an infusion reaction, characterized by fever, chills, rash, swelling (of hands, feet, and face), bronchospasm, and hypotension. In most cases, the reaction is immediate (30 minutes to 2 hours), usually during the first infusion, but is less severe with subsequent infusions. Pretreatment with acetaminophen and an anti-histamine is recommended to prevent this infusion reaction. If the infusion reaction occurs, the infusion rate should be decreased or discontinued. Additional treatment with steroids may also be warranted. Rituximab treatment requires monitoring of several AEs, including infections, TB, and lymphoma [22]. It is contraindicated in the case of pregnancy and breastfeeding, active infections, live vaccination, severe congestive heart failure, a history of demyelinating disease, and a 5-year history of non-lymphoproliferative cancer [23].

In a meta-analysis assessing the safety of rituximab, including long-term therapy, 123 of 2,578 patients with RA withdrew because of malignancy, infection, a severe infusion reaction, or cardiac event [22]. Most AEs occurred during the first course of therapy. The incidence of malignancies was not increased in patients with RA treated with rituximab [22].

Rituximab causes a decrease in gammaglobulin concentrations, depending on the cumulative dose; however, this does not seem to lead to a higher risk for severe infection [23]. A few cases of progressive multifocal leukoencephalopathy (PML) were reported in some patients after rituximab therapy [22].

### Ofatumumab

#### Mechanism

Ofatumumab (trade name Arzerra) is a fully human monoclonal antibody directed against the membrane proximal epitope on the CD20 molecule (Table 1) [24,25].

#### Indications and dosage

Ofatumumab is indicated for the treatment of chronic lymphocytic leukemia. Owing to its B-cell-suppressing effect, it is also used in the USA and Europe as an off-label treatment for patients with RA who have failed MTX therapy (Table 1). In a combined phase I and II study, patients received three escalating doses of ofatumumab (300, 700, and 1000 mg), with each dose given as two separate IV administrations with 2 weeks between them, over a period of 24 weeks [25]. Prior to administration, all patients in the high-dose groups received premedication with acetaminophen, anti-histamine, and glucocorticoids.

The recommended protocol for maximum efficacy and safety is IV administration of 700 mg over 4 hours with appropriate premedication, repeated every 2 weeks [24,25].

#### Efficacy

Two studies reported efficacy of ofatumumab compared with placebo or MTX in patients previously treated with DMARDs or biologic therapies; all such therapies were discontinued prior to study entry [24,25]. In one study, comparing the efficacy of ofatumumab (three different dose groups) with patients treated with placebo or MTX, significantly higher rates of ACR20 were seen in the ofatumumab groups compared with the placebo group. In addition, the efficacy was dose-dependent, as assessed by the ACR20 and the circulating B-cell population [24].

In another multi-center double-blind RCT of biologic therapies in naive patients with RA, ofatumumab 700 mg was compared with placebo, and a significantly higher rate of improvement as measured by ACR20 was noted in the ofatumumab group [25]. No significant differences were noted between seronegative and seropositive patients in this study [25].

#### Adverse effects and safety

In a study comparing the safety of ofatumumab (three different dose groups), the main AEs were related to infusion reaction, which was mild to moderate and occurred mainly with the first and second administrations [24]. However, after 24 weeks of treatment, a significant number of AEs occurred in the highest-dose (1000 mg) group [24]. No difference in infections was noted between the different dosage groups and the placebo [24]. Other AEs included rash, dyspnea, rhinitis, nausea, pruritus, URT infections, headaches, fatigue, flushing, hypertension, and diarrhea.

In another double-blind multi-center study, the most common reactions were urticaria and rash on the day of the first infusion. Most of the reactions were mild to moderate, and severe AEs were infrequent [25]. No case of PML was reported [25].

### Belimumab

#### Mechanism

The BLyS protein is a member of the superfamily of TNFs. It inhibits B cell apoptosis and stimulates the differentiation of B cells into immunoglobulin-producing plasma cells. Belimumab (trade name (Benlysta) is a human monoclonal immunoglobulin (IgG1γ), which binds to and inhibits the soluble form of the BLyS protein [26,27] (Table 1).

#### Indications and dosage

Belimumab is approved by the FDA for the treatment of mild to moderate SLE, and is presently not indicated for active LE nephritis or neuropsychiatric involvement (Table 1) [26,27]. It is given by slow IV infusion over 1 hour at a recommended dose of 10 mg/kg at 2-week intervals for three cycles, then once every 4 weeks [26,27].

#### Efficacy

In a phase II trial, there was a significant effect of belimumab after 52 weeks of treatment and a steroid-sparing effect. However, no improvement in the SLE responder index (SRI), which incorporates disease activity scores to compile a single score, disease activity score was significant in SLE patients with severe active disease [26]. In the phase III clinical trials assessed using the SRI, a significant improvement was seen in both the 1 mg/kg and the 10 mg/kg groups compared with the placebo group [28]. Significant changes were also reported in the SELENA-SLEDAI (Safety of Estrogens in Lupus Erythematosus National Assessment–Systemic Lupus Erythematosus Disease Activity Index) trial, with non-inferiority of belimumab compared with placebo, as measured by the Physician’s Global Assessment (PGA) [26].

#### Adverse effects and safety

AEs associated with belimumab treatment include nausea, diarrhea, headaches, and URT infections. Less common side effects are fever, cystitis, leucopenia, infusion reaction, and severe infections. Studies have shown that the number of AEs associated with belimumab were similar to that in the placebo group, and that the severity and number of AEs did not increase in the high-dose (10 mg/kg) group compared with the low-dose (1 mg/kg) group [26,27].

### Epratuzumab

#### Mechanism

Epratuzumab is an IgG1 monoclonal antibody directed against the CD22 molecule. CD22 is a B-cell-specific transmembrane sialoglycoprotein that inhibits the B-cell receptor complex, causing early apoptosis and thus shortening the cell’s life span (Table 1) [28,29].

#### Indications and dosage

The therapeutic dose of epratuzumab is 360 mg/m^2^ IV over 1 hour every 2 weeks for up to four cycles. It is recommended that acetaminophen and anti-histamine should be administered prior to infusion to minimize infusion reaction [30]. Owing to its anti-CD22 targeting activity, epratuzumab is indicated for the treatment of SLE and Sjögren’s syndrome (Table 1) [30].

#### Efficacy

Although epratuzumab has not received regulatory approval for SLE and Sjogren's syndrome, several studies have assessed the drug for these two conditions (excluding patients who were previously treated with rituximab). In a study measuring scores on the British Isles Lupus Assessment Group (BILAG) scale during a follow-up of 6 months, all patients had a decrease in disease activity of more than 50% [30]. In a larger study of 227 patients with moderate to severe SLE, comparing doses of 600, 800, 2,400, or 3,600 mg epratuzumab with placebo for 12 weeks, all epratuzumab groups had a significant response as measured by BILAG score [29]. In a different study of patients with Sjögren’s syndrome, 67% of patients responded to 6 months of epratuzumab therapy, indicating regeneration of glandular tissue [30].

#### Adverse effects and safety

In the phase II trial of 227 patients described above, there was no significant difference in AEs between the placebo group and any of the epratuzumab groups [30].

In the study of patients with SLE, most of the AEs were minor, and included nausea, fatigue, general pain, and infusion reaction [30]. Patients with Sjögren’s syndrome had more severe AEs during the infusion, including swelling of the nasal mucosa and increased pressure in the glottis [30].

### Abatacept

#### Mechanism

T cells play a major role in the pathogenesis of RA. Co-activation of CD28 with the antigen-presenting cell (APC) protein CD80/86 results in release of inflammatory cytokines. Cytotoxic T lymphocyte-associated (CTLA)-4 is a protein with a high affinity to CD80/86, which inhibits T-cell activation by blocking the CD28 binding. Abatacept (trade name Orencia) is a CTLA-4 IgG1 that binds to CD80/86 on APCs, inhibiting the co-stimulation of CD28 on T cells (Table 1) [31].

#### Indications and dosage

Abatacept is approved by the FDA for the treatment of RA that is non-responsive to DMARDs and anti-TNF-alpha blockers, and for JIA (Table 1). For RA treatment, the dose is based on body weight, with a dose of approximately 10 mg/kg (patients weighing less than 60 kg receive 500 mg; those weighing 60 to 100 kg received 750 mg, and those weighing more than 100 kg receive 1000 mg). The initial IV dose can be repeated by additional doses after 2 and 4 weeks, with further doses every 4 weeks after that. Another treatment option after the initial IV dose is to administer a subcutaneous (SC) injection of 125 mg at 24 hours after the first infusion, repeated by weekly SC injections of 125 mg [31,32].

For JIA, the route of administration is also IV, and the dose is based on body weight and age (children 6 years over weighing less than 75 kg receive 10 mg/kg, those weighing 75 to 100 kg receive 750 mg, and those weighing over 100 kg receive 1000 mg).

The same protocol as described above for patients with RA was used in a multi-center double-blind placebo-controlled study of 180 patients with SLE with discoid rash, serositis, or polyarthritis. The results of the study suggest some abatacept efficacy in patients with non-life-threatening manifestations of SLE, but due to safety issues, further assessment.is warranted [32].

#### Efficacy

In a meta-analysis comparing phase II and III studies including an extension phase, improvement in disease activity in patients with RA, as measured by the ACR score, was reported with combined therapy with abatacept and DMARDs within 6 months. This included patients who had not responded to prior anti-TNF-alpha therapy. There was an average improvement of 50% in ACR20 in the abatacept group, compared with 30% in the placebo group. Similar results were found for ACR50 and ACR70 in the abatacept group compared with the placebo group and with other treatment groups [33]. In a long-term study comparing abatacept with placebo (with background therapy for both groups of a steady dose of MTX), the response to abatacept was superior and was maintained for 3 years including physical function scores [34]. Evaluation of radiographic changes identified a reduction in bone erosion scores (Genant-modified Sharp scoring method) every year within the 3-year follow-up, and 40% of patients had no radiographic progression after 3 years [35].

In another study of 180 patients with SLE, abatacept had a steroid-sparing effect and resulted in a lower rate of flares. This effect was predominant in the lupus patients with polyarthritis [32]. Furthermore, in the abatacept group, significant improvement in well being was reported by the HAQ (Health Assessment Questionnaire) [32].

#### Adverse effects and safety

The main AEs caused by abatacept are infections, URT symptoms, nausea, headache, infusion reaction, fever, hypertension, and back and limb pain.

In a study of long-term safety, 96% of patients experienced AEs; however, most of them were mild to moderate and mostly related to infections. No significant differences in AEs were noted when the abatacept group and the placebo group (receiving DMARDs only) were compared [35].

### Novel anti-TNF-α blockers

#### Golimumab

##### Mechanism

Golimumab (trade name Simponi) is a fully human monoclonal IgG1 antibody, acting on both soluble and membrane-bound TNF-α (Table 1) [36].

##### Indications and dosage

Golimumab is approved by the FDA for RA, psoriatic arthritis (PsA), and ankylosing spondylitis (AS) (Table 1) [37-40]. The indicated dose for all three diseases is 50 mg monthly by SC injection. For RA, golimumab is administered in combination with MTX; for PsA it may be administered alone or with MTX; and for AS, it may be administered alone [40].

In a study comparing treatment groups given doses of 50 or 100 mg golimumab SC, no significant difference occurred between the two groups. The lower dosage is recommended by the FDA [37].

##### Efficacy

In a study of patients with AS comparing different doses of golimumab with placebo for 24 weeks, a significant improvement was found in the AS International Working Group criteria (20% improvement; ASAS20) for all golimumab groups compared with the placebo group [37]. In a similar study in patients with PsA, using two different doses of golimumab; a significant improvement was encountereed in both golimumab groups compared with placebo [37]. Furthermore, there was a significant improvement in both enthesitis and Dactylitis Severity Score [37].

In a study of patients with RA who were not responsive to MTX, (GO-FORWARD study [38]) the efficacy of MTX plus placebo, MTX plus golimumab 50 mg, MTX plus golilumab 100 mg, or golimumab 100 mg plus placebo, were compared. The most significant results were seen in the MTX plus golimumab groups (both doses) compared with MTX alone. However, a higher incidence of AEs was noted in the golilumab 100 mg group [38]. In another study (GO-BEFORE study [39]), using the same protocol as the previous study but in MTX naïve patients with RA, a significant response was seen with golimumab as measured by ACR70, ACR90, 28-item Disease Activity Score (DAS28), and HAQ [39]. The primary outcome of ACR50 was not achieved, which could be attributable to lower disease activity in these patients compared with patients receiving other biologic therapy-based studies, as well as to group size and possibly C-reactive protein levels. However, the modified intention-to-treat analysis of the primary endpoint and other pre-specified efficacy measures did show efficacy in both the golimumab plus MTX groups in these patients [39].

##### Adverse effects and safety

The main AEs are infections (mostly of the URT) and nausea. Additional AEs include hypertension, abnormal liver function (patients with latent TB were required to take prophylactic treatment, resulting in greater liver abnormalities), paresthesia, dizziness, constipation, local skin reaction, and some cases of malignancy (basal and squamous cell carcinomas, and prostate, lung, and breast cancers) were reported [37-40].

In studies performed to assess golimumab safety, more infections occurred in patients receiving golimumab compared with those receiving placebo [36-39]. In the study on patients with PsA, those who received 100 mg golilumab had significantly higher rates of infections than those who received 50 mg, but these were mainly minor infections involving the URT [38].

#### Certolizumab pegol

##### Mechanism

Certolizumab (trade name Cimzia) is a pegylated Fab fragment of a humanized TNF-alpha monoclonal antibody, which binds to and inhibits TNF-alpha. The pegylation extends the half-life of the antibody, and the missing Fc fragment reduces the risk of cytotoxicity (Table 1) [41,42].

##### Indications and dosage

Certolizumab is approved by the FDA for the treatment of active RA disease (Table 1), given as SC injection of 400 mg every 2 weeks for three consecutive cycles, followed by maintenance therapy of 200 mg SC every 2 weeks.

##### Efficacy

In a study of patients diagnosed with RA for no less than 6 months and no more than 15 years, who had not received any biologic therapy for 6 months before the beginning of the study, but had responded to anti-TNF-alpha blockers in the past, were enrolled. The first group was treated with MTX plus placebo and the second with MTX plus certolizumab. ACR20 response, physical improvement, and reduction in radiographic progression were achieved more rapidly in the certolizumab group compared with the placebo group over a 1-year period [41].

In a different study of patients with RA who had experienced treatment failure with DMARDs, a significant ACR20 response of up to 50% was accomplished with certolizumab monotherapy. Similar improvements were also encountered for disease activity, physical function, and arthritic pain [42].

##### Adverse effects and safety

Other than the AEs already recognized for treatment with TNF-alpha blockers, a higher incidence of serious infections was noted with certolizumab [42]; these AEs occurred with the lower dose of 200 mg but not the higher dose of 400 mg [42].

In a safety study of patients with RA, the AEs reported for certolizumab were headache, nasopharyngitis, diarrhea, and sinusitis [42]. A significantly higher incidence of serious AEs were detected in patients receiving certolizumab, and these included bacterial arthritis, salmonella arthritis, ischemic stroke, menorrhagia, mastitis, and increases in blood creatinine and urea levels [42]. There were no fatalities or cases of drug-induced SLE [42].

#### Sifalimumab

Sifalimumab is an anti-interferon (IFN)-alpha monoclonal antibody. Patients with SLE carry a typical type I IFN signature. Compared with normal controls, overexpression of B cell activation factor (BAFF) mRNA in whole blood characterizes patients with SLE. The effect of anti-IFN-alpha antibody was examined using B lymphocyte stimulator/BAFF, and PCR identified suppression of BAFF mRNA (Table 1) [43,44].

The safety of sifalimumab was evaluated in a phase I trial of SLE. There were no drug-related AEs and no increase in viral infections. In the trial, there was improvement in disease activity with sifalimumab compared with placebo [44], and the drug is currently in phase III trials [20].

### Interleukin-1 inhibitors

Anakinra (trade name Kineret) is approved by the FDA for treatment of RA. Recently, other IL-1 blockers became available. Two of these are indicated for the treatment of disorders collectively known as cryopyrin-associated periodic syndromes (CAPS).

Canakinumab (trade name Ilaris) is specifically indicated in adults and in children aged 4 years and older for the treatment of CAPS, including familial cold auto-inflammatory syndrome (FCAS) and Muckle-Wells syndrome (MWS) (Table 1). For adults, it is administered via SC injection at a dose of 150 mg every 8 weeks. Canakinumab is beneficial, safe, and leads to a reduction in serum amyloid A (SAA) and C-reactive protein (CRP) levels. The associated AEs include infections [45].

Rilonacept (trade name Arcalyst) is an IL-1 blocker that is also indicated for the treatment of CAPS, including FCAS and MWS, in adults and in children aged 12 years and older. Rilonacept (160 mg SC weekly for adults and 2.2 mg/kg or up to 160 mg for children) maintained long-term efficacy and safety in an extension study of 72 to 96 weeks, including normalization of the inflammatory markers CRP and SAA. AEs were mild to moderate, and included injection site reactions and URTI [46]. IL-1 inhibitors are currently under investigation for the treatment of acute gout flares [47].

### Intravenous immunoglobulin therapy

#### Mechanisms

IVIG is a well established therapy for immunodeficiency diseases, and is beneficial for autoimmune diseases [48]. The immunoglobulins are derived from a pool of thousands of healthy donors, containing antibodies to both self and foreign antigens.

Several mechanisms of action are suggested for immunoglobulins, and they influence the immune system at many levels. IVIG has a direct neutralization effect on pathogenic antibodies, B and T cells, and macrophage regulation. It inhibits the differentiation and maturation of dendritic cells, prevents the presentation of self antigens, modulates IL-1 receptor antagonists, and inhibits BLyS activity (Table 1) [49].

#### Dosage protocols

The characteristics of the various IVIG products are not discussed here, but can be readily found online (http://www.medprorx.com/ref_ivig_drug.html). Different IVIG protocols are in use, depending on the disease. In systemic autoimmune diseases such as SLE, a high-dose protocol is often used, consisting of 2 g/kg divided over 5 days. This is repeated every 4 weeks, a period that allows the immunoglobulin level in the serum to return to normal. The cycle repeats every month, usually for up to 6 months, and then every 2 to 3 months if required. Long term, therapy remains beneficial [50]. To reduce AEs, a single dose of hydrocortisone 100 to 200 mg should be administered on day 1 before initiation of the IVIG therapy. Treatment with a single dose of low-molecular-weight (LMW) heparin before IVIG initiation may prevent possible thromboembolic AEs [51]. Low-dose IVIG therapy (400 mg/kg over 1 day every 3 to 4 weeks) is beneficial for organ-specific disease, predominantly neurological conditions [50]. It may also be useful for some cases of mild to moderate SLE [52].

#### Efficacy

IVIG may be an adjunctive therapy for patients with SLE who are refractory to conventional therapies. It may also be an option for young women who do not wish to risk the problems of disfigurement or sterility associated with conventional immunosuppressive therapies. Based on the various case reports and case series, it appears that high-dose IVIG is beneficial for moderate to severe SLE. It is beneficial for the treatment of serositis, cardiopulmonary disease, hematological conditions, diffuse neuropsychiatric disease, and lupus nephritis [53-55]. In patients with SLE, IVIG has a steroid-sparing effect [56]. High-dose IVIG was beneficial for SLE, and led to a decrease in various scores of disease activity. IVIG may be useful for recalcitrant cases of organ-specific cutaneous LE including discoid LE and subacute cutaneous LE. In many cases, patients received short-term therapy with moderate or low doses [48]. IVIG causes a serological improvement including a decrease in antibody titer levels and an increase in complement levels [49].

IVIG may be beneficial as off-label for a number of other autoimmune diseases including Sjogren's syndrome, polyneuropathy, severe PV, Still’s disease, and relapsing anti-neutrophil cytoplasmic antibody-associated vasculitis [49]. Low-dose IVIG is beneficial for Guillain–Barré syndrome, Lambert–Eaton syndrome, refractory myasthenia gravis, refractory multiple sclerosis, multifocal motor neuropathy, chronic inflammatory demyelination polyneuropathy, dermatomyositis, and stiff person syndrome [48]. In addition, polyneuropathy in SLE and vasculitis respond well to IVIG therapy [57]. Other diseases in which IVIG is used include refractory uveitis, Graves' disease, and ocular involvement in Behcet's disease [48].

#### Adverse effects and safety

IVIG is usually associated with mild and transient AEs. The common mild AEs include arthralgia, myalgia, weakness, abdominal pain, diarrhea, chills, dizziness, drowsiness, fatigue, fever, headache, and changes in blood pressure or heart rate [50]. The more severe AEs include anaphylactic reaction, thromboembolic events, neutropenia, pancytopenia, autoimmune hemolytic anemia, renal failure with acute tubular necrosis, asthma exacerbation, hepatic dysfunction, seizures, acute respiratory distress syndrome, and aseptic meningitis [55,58]. AEs occurring with the first course may return with further courses [50], and do not usually worsen with long-term IVIG therapy [50].

Special attention is advised when treating patients with pro-thrombotic risk and those with renal failure or who are at risk of renal tubular injury (dehydration, renal disease, diabetes). In cases where there is a high risk of thromboembolic event, LMW heparin is to be administered before initiation of the IVIG course. IVIG is contraindicated in patients with IgA deficiency [49].

### Immunogenicity of biologic therapies

Biologic agents are engineered molecular targeted therapies, which include antibodies and receptor blockers. Antibodies may be chimeric or fully human. As a result, patients may develop immunogenicity to these agents, characterized as development of antibodies to the biologic agent itself, or the development of pathogenic autoantibodies that can lead in some cases to the development of another autoimmune disease. Antibodies targeted against the biologic therapy, termed human anti-chimeric antibodies (HACA), may lead to a decrease in the efficacy of the medication, hence a DMARD (usually MTX) is often recommended as co-treatment to counteract the increased levels of HACA. With the availability of fully human antibody biologic agents, the development of HACA may be negligible. Although the development of antinuclear antibodies and anti-double-stranded DNA antibodies have been reported secondary to biologic treatment, the development of frank SLE is infrequent. However, drug-induced SLE secondary to biologic therapies is not a mild disease, as usually seen for other medications, but rather a severe systemic disease with renal impairment [59].

### Immunogenicity of specific drugs

The newly developed drugs may not cause significant immunogenicity effects. Several studies of TCZ showed that the development of anti-chimeric antibodies had no significant clinical effect [5].

In a study of 130 patients receiving ofatumumab, no patients developed drug antibodies [25], and in a study of belimumab treatment, negative seroconversion occurred significantly more frequently in the belimumab treatment group [27].

In a trial comparing the immunogenicity of epratuzumab with rituximab, fewer anti-chimeric antibodies developed compared to epratuzumab [29]. In a study of 339 patients receiving abatacept, only two patients developed drug-related antibodies, which was not statistically significant [32]. In the GO-FORWARD study of golimumab, only 2.1% of patients with RA developed drug antibodies [39].

In a phase I multi-center double-blind trial of 33 patients treated with sifalimumab, there were no anti-sifalimumab antibodies detected prior to administration or at several points during the study [44]. In a study of 619 patients treated with certolizumab, only 5.1% of patients developed anti-drug antibodies, which was not statistically significant [42].

## Discussion

We reviewed the literature on new biologic therapies that have recently become available and how they are used to treat various autoimmune diseases. Some of these are new drugs in known classes (TNF alpha blockers and B cell modulators),whereas others are of a new class (BLyS inhibitors, IFN I inhibitors, IL-1 inhibitors). The AEs are similar for all the biologic therapies reviewed, with the severe AEs including serious infections. TB recurrence has been virtually eradicated due to the accepted screening recommendations before starting biologic therapy.

It is advantageous to have a range of effective biologic drugs available for patients with severe or resistant disease. Severe disease can include involvement of vital systems, persistent disease despite conventional therapy or even persistent and non-responsive disease despite biologic therapies. Different drugs from the same class (for example anti-TNF-alpha blockers) may provide further treatment options. If a patient develops an AE from one TNF-alpha blocker, they can be switched to a different TNF alpha blocker or to another class of drugs (for example, B-cell modulators). Furthermore, if there was an initial efficacy response that ceased over time, a switch to a different biologic class may achieve a favorable result. With the various available biologic therapies, the therapy can be tailored to the individual patient. For example, IV therapy may be suitable for patients who need medical supervision, whereas SC therapy may be suitable for patients who feel confident injecting the drug in the privacy of their own home. In addition, the choice of biologic may be customized to include considerations of co-morbidities or the need for concomitant drug treatment. There is a focus on early treatment, yet none of the biologic therapies is yet available as first-line medication for autoimmune diseases, probably due to economic concerns. Because the complexity of preparing biologic drugs has diminished over the years, costs may be driven down, allowing biologic therapies to be used at an early stage of disease and hence enable prevention of irreversible damage.

## Conclusions

The arsenal of biologic therapies available to treat autoimmune diseases is quickly expanding as a result of better understanding of molecular mechanisms together with improved production capacity. They include (by class): novel anti-TNF alpha blockers (fully humanized or pegylated), anti-IL agents (to IL-1, IL-6), B-cell-directed therapies (to CD20, CD22), co-activation signaling (CTLA4-Ig), and IVIG. Although most of the FDA-approved biologic therapies are for RA, Belimumab is the first FDA-approved targeted therapy for SLE. In addition, the efficacy and safety for biologics in off-label indications are encouraging for patients with resistant autoimmune conditions.

## Abbreviations

ACR: American College of Rheumatology; Anti-TNF: Tumor necrosis factor antagonist; AS: Ankylosing spondylitis; ASAS: Assessment in Ankylosing Spondylitis; BLySS: B lymphocyte stimulator; BILAG: British Isles Lupus Assessment Group; CRP: C-reactive protein; DAS28: 28-item Disease Activity Score; DMARD: Disease-modifying anti-rheumatic drug; EC50: Half-maximal effective concentration; GI: Gastrointestinal; HAQ: Health Assessment Questionnaire; IFN: Interferon; ITP: Immune thrombocytopenic purpura; IVIG: IV immunoglobulin; JIA: Juvenile idiopathic arthritis; MTX: Methotrexate; MPA: Microscopic polyangiitis; RA: Rheumatoid arthritis; RCT: Randomized controlled trial; SAA: Sserum amyloid A; SELENA-SLEDAI: Safety of Estrogens in Lupus Erythematosus National Assessment-Systemic Lupus Erythematosus Disease Activity Index; SJIA: Systemic juvenile idiopathic arthritis; TB: Tuberculosis; TCZ: Tocilizumab; TNF: Tumor necrosis factor-α; URT: Upper respiratory tract.

## Competing interests

The authors do not have any competing interests.

## Authors' contributions

RZ and ZGG contributed equally to the literature search, review of the pertinent articles, and writing of the article. SY contributed to the concept, editing, and critical appraisal of the article. All authors read and approved the manuscript for publication.

## Pre-publication history

The pre-publication history for this paper can be accessed here:

http://www.biomedcentral.com/1741-7015/11/88/prepub
